# Amyloidogenic Propensity of a Natural Variant of Human Apolipoprotein A-I: Stability and Interaction with Ligands

**DOI:** 10.1371/journal.pone.0124946

**Published:** 2015-05-07

**Authors:** Silvana A. Rosú, Omar J. Rimoldi, Eduardo D. Prieto, Lucrecia M. Curto, José M. Delfino, Nahuel A. Ramella, M. Alejandra Tricerri

**Affiliations:** 1 Instituto de Investigaciones Bioquímicas de La Plata (INIBIOLP), CONICET, La Plata, Buenos Aires, Argentina; 2 Facultad de Ciencias Médicas, Universidad Nacional de La Plata, La Plata, Buenos Aires, Argentina; 3 Instituto de Investigaciones Fisicoquímicas Teóricas y Aplicadas (INIFTA), Universidad Nacional de La Plata-CONICET, La Plata, Buenos Aires, Argentina; 4 Departamento de Química Biológica e Instituto de Bioquímica y Biofísica, Facultad de Farmacia y Bioquímica, Universidad de Buenos Aires, Buenos Aires, Argentina; University of South Florida College of Medicine, UNITED STATES

## Abstract

A number of naturally occurring mutations of human apolipoprotein A-I (apoA-I) have been associated with hereditary amyloidoses. The molecular mechanisms involved in amyloid-associated pathology remain largely unknown. Here we examined the effects of the Arg173Pro point mutation in apoA-I on the structure, stability, and aggregation propensity, as well as on the ability to bind to putative ligands. Our results indicate that the mutation induces a drastic loss of stability, and a lower efficiency to bind to phospholipid vesicles at physiological pH, which could determine the observed higher tendency to aggregate as pro-amyloidogenic complexes. Incubation under acidic conditions does not seem to induce significant desestabilization or aggregation tendency, neither does it contribute to the binding of the mutant to sodium dodecyl sulfate. While the binding to this detergent is higher for the mutant as compared to wt apoA-I, the interaction of the Arg173Pro variant with heparin depends on pH, being lower at pH 5.0 and higher than wt under physiological pH conditions. We suggest that binding to ligands as heparin or other glycosaminoglycans could be key events tuning the fine details of the interaction of apoA-I variants with the micro-environment, and probably eliciting the toxicity of these variants in hereditary amyloidoses.

## Introduction

Amyloidoses are a large group of heterogeneous diseases characterized by insoluble proteins inducing organ damage [1]. Many precursor proteins are associated with these pathologies, and numerous studies have shown that the ability to form amyloid fibrils is an inherent property of the polypeptide chain sequence. In this respect, different hereditary amyloidoses are due to single point mutations which increase the pathological behavior of the affected protein. Nevertheless, the reason by which the pathway to pathogenicity occurs seems to depend on a large number of factors either intrinsic to the protein sequence-conformation or to the local micro-environment in which proteins exert their function. Moreover, a large body of research indicates that, in addition to the fibers that are the signature of this disease, more disorganized and soluble species could modulate the cytotoxicity of the misfolded protein conformations [2,3].

Human apolipoprotein A-I is the major protein among high density lipoproteins (HDL), associated with key functions preventing atherosclerosis. Extensive research supports that the cardioprotection attributed to the protein is due to its active role in the reverse cholesterol transport pathway and in the protection against endothelial dysfunction [4–6]. In order to fulfill those functions, it was speculated that the *in vivo* efficiency of apoA-I strongly depends on its ability to dissociate from spherical HDL giving rise to stable, lipid-poor apoA-I, which can be rapidly lipidated as a consequence of its interaction with cellular macrophages ATP binding cassette A1 (ABCA1) transporter [7]. Nevertheless, the structural disorder of the lipid-free conformation represents a potential risk of self-aggregation. About 20 mutations in human apolipoprotein A-I (apoA-I) have been found associated with hereditary systemic amyloidoses [8] and it is probable that there are more sequences that have not yet been described, as genetic studies of hereditary pathologies are not usually performed in underdeveloped countries. Eriksson et al. (2009) have identified two ‘hot spots” in apoA-I sequence in which most of the mutations are described [8]. About 50% of the mutants involve substitution of residues in the region spanning from amino acid 50 to 93, mainly causing patients to suffer from hepatic or renal amyloidoses [9,10]. On the other hand, a large number of mutations point to the stretch between residues 173 to 178, mainly triggering amyloidoses of the heart, larynx or skin. Nevertheless, in spite of the location of the mutation, proteolysis between residues 83 and 93 is involved in apoA-I amyloidosis, resulting that the N-terminus is the predominant form of the protein found in amyloid fibril deposits in patients`lesions [11].

In a previous report, we have studied two natural variants, a deletion mutant Lys107-0, inducing amyloidoses associated to severe atherosclerosis [9] and Gly26Arg, associated with polyneuropathy [12]. We have identified common and specific events that could mediate the misfolding of mutants and pathology [13]. Taking advantage of the lessons learned, we moved forward to understand the consequences of the Arg173Pro mutation, in patients with cardiopathy and skin compromise [14]. We have assayed structural parameters of the target protein and postulate that the interaction with natural ligands could play-at least in part- an important role in the pathological pathway caused by this mutant.

## Materials and Methods

Guanidine hydrochloride (GndHCl), sodium dodecyl sulfate (SDS) and thioflavin T (ThT) were from Sigma-Aldrich (St Louis, MO); 1,2-dimyristoylsn-glycero-3-phosphatidylcholine (DMPC) was from Avanti Polar Lipids (Alabaster, AL). His-purifying resin was from Novagen (Darmstadt, Germany). Heparin (for clinical application) from bovine intestinal mucosa (average molecular weight 15 kDa) was from Rivero (BA, Argentina). 4,4’-dianilino-1,1’-binaphthyl-5,5’-disulfonic acid, dipotassium salt (bis-ANS) was purchased from Molecular Probes (Invitrogen, Carlsbad, CA). All other reagents were of the highest analytical grade available.

### Cloning, expression and purification of wild-type (wt) and Arg173Pro mutant apoA-I

The cDNA for human apoA-I, kindly donated by Dr A. Jonas (University of Illinois at Urbana-Champaign, IL), was further modified to introduce an acid labile Asp—Pro peptide bond between amino acid residues 2 and 3 of apoA-I, which allowed specific chemical cleavage of an N-terminal His-Tag fusion peptide [15,16]. This construct, inserted into a pET-30 plasmid (Novagen, Madison, WI), was used to express wt apoA-I, and we have previously checked that the protein behaved indistinguishably from plasma apoA-I isolated from healthy donors [16]. Next, it was used as a template for the construction of the Arg173Pro mutant. The mutation was introduced using the Quickchange method (Stratagene, La Jolla, CA). The sequence for the coding strand of the mismatch primer was the following: 5`- GAGCTGCGCCAGCCCTTGGCCGCGCG-3`; Protein expression and purification were performed as previously described [13,15].

### Protein denaturation and stability

ApoA-I wt and Arg173Pro were diluted in citrates phosphates McIlvaine`s buffer [17], either at pH 7.4 or 5.0. Intrinsic fluorescence emission spectra (corresponding to the average signal from four naturally occurring Trp residues: position numbers 8, 50, 72 and 108 in the primary sequence of the native protein) were measured at 25°C in an Olis upgraded SLM4800 spectrofluorometer (ISS Inc, Champaign, IL), with excitation set at 295 nm, as described elsewhere [15]. Chemical denaturation was performed by incubation of 0.1 mg/mL apoA-I variants in the presence of increasing concentrations of GndHCl. In order to correlate the characterization of this mutant with other pro-amyloidogenic variants and the wt protein described before [13,15], the free energy of unfolding in the absence of denaturant (ΔG°) was calculated from the shift in the spectral center of mass of the fluorescence emission, assuming a two-state process, as previously described [18,19]. As we are aware that this parameter and also the maximum wavelength of fluorescence emission [20] hold a non-linear relationship with the molar fraction of native-or unfolded- protein, the evolution of the emission intensity at 335 nm as a function of GndHCl concentration was also analyzed (not shown). Results derived from this analysis are in good agreement with those presented in this paper. Alternatively, denaturation was monitored by incubating 0.1 mg/mL apoA-I variants with the fluorescence probe bis-ANS at a 1:1 (probe:protein) molar ratio and measuring emission spectra after stepwise addition of GndHCl.

### Protein structure under native conditions

Solvent exposure of Trp residues was determined by fluorescence quenching induced upon increasing concentrations of acrylamide, as previously described [21] [15]. Proteins were diluted to a final concentration of 0.1 mg/mL, and quenching measured after stepwise addition of acrylamide. The recovered parameter K is the quenching constant.

The presence of exposed hydrophobic domains in the native structure of apoA-I variants was assessed by titration with bis-ANS [22,23]. Small aliquots of bis-ANS (from a concentrated stock solution in methanol) were added to 0.1 mg/mL apoA-I variants at 25°C and fluorescence emission spectra acquired between 450–550 nm with excitation set at 395 nm. Residual methanol concentration was kept to a minimum in order to avoid structural artifacts due to solvent effects.

Circular dichroism (CD) spectra were registered on 0.1 mg/mL solutions of proteins keeping McIlvaine`s buffer to a minimum to avoid the interference of high salt concentration and excessive optical background. Spectra were recorded on a Jasco J-810 spectropolarimeter. Data in the far UV (200–250 nm) region was collected using a 1 mm path-length cuvette. A scan speed of 20 nm min^-1^ with a time constant of 1 s was used for both wt and Arg173Pro mutant proteins. Each spectrum was measured at least three times and data were averaged to minimize noise. Molar ellipticity was calculated as described elsewhere [24], using a mean residue weight value of 115.5 and 115.2 for wt and Arg173Pro proteins, respectively.

### Fluorescence Resonance Energy Transfer of Trp to bis-ANS

Fluorescence resonance energy transfer (FRET) is a distance-dependent interaction between the electronic excited states of two fluorescent molecules, in which excitation is transferred from a donor (D) to an acceptor molecule (A), without emission of a photon. The efficiency of energy transfer (E) can be estimated by the equation:

$$\text{E}=\frac{\;Ro^{6}}{\left(Ro^{6}+Rd^{6}\right)}$$ (1)

where R_o_ represents the Förster radius, the distance where the transfer is 50% efficient (for this donor-acceptor pair a distance of 26,3 Å is calculated [25]), and R_d_ is the actual distance between donor and acceptor. When the donor and acceptor are chemically different, FRET is typically measured using the relative fluorescence intensity of the donor in the presence (F_DA_) and in the absence (F_D_) of the acceptor [21]:

$$E=\frac{1-F_{DA}}{F_{D}}$$ (2)

In order to determine fluorescence energy transfer, from Trp (donor) to bis-ANS (acceptor), 0.1 mg/mL of the Arg173Pro protein was incubated either at pH 7.4 or 5.0 and the Trp emission intensity registered, with excitation set at 295 nm and the emission recorded between 300 and 550 nm. Next, the same spectrum was measured after the addition of 1.6 μM bis-ANS. Direct excitation of bis-ANS was achieved at 395 nm.

### Detection of protein aggregates

ApoA-I (0.2 mg/mL) variants were incubated at different pH values. After 48 h at 37°C, thioflavin T (ThT) was added at a 1:1 molar ratio and fluorescence intensity was measured on a Beckman Coulter DTX 880 Microplate Reader (Beckman, CA), using excitation and emission filters centered at 430 nm and 480 nm, respectively. When indicated, light scattering was also detected with the spectrofluorometer, setting both excitation and emission wavelengths at 350 nm.

### DMPC clearance assay

Multilamellar liposomes (MLV) were prepared from a stock solution of DMPC in chloroform. Lipids were dried under N_2_ atmosphere and resuspended in Tris 20 mM buffer pH 7.4 by exhaustive vortexing at 37°C [16]. Proteins, at a final concentration of 7 μM in the same buffer were incubated at 24°C with DMPC MLV, at a molar ratio lipid:protein 40:1 and absorbance monitored by 90 min using a 350 nm filter on the Microplate Reader. The final products after 1 and 2 h incubation were analyzed by polyacrylamide gradient gel electrophoresis under non-denaturing conditions (PAGGE).

### Binding of ligands to apoA-I variants

Wt and Arg173Pro (0.2 mg/mL) proteins were incubated with 0.2 mM SDS (either at pH 7.4 or 5.0) for 48 h at 37°C. Complex formation was detected by the binding to ThT as described above. Protein binding of heparin was tested following incubation of 0.2 mg/mL proteins with heparin at a 2:1 heparin/protein molar ratio for 48 h at 37°C. In order to confirm whether binding to heparin results in the formation of high molecular weight complexes, the incubation mixtures were analyzed by PAGGE. In a separate experiment, the Arg173Pro variant was incubated at 0.5 mg/mL and pH 7.4 for 24 h at 37°C in the absence or in the presence of heparin, and morphology was compared by atomic force microscopy (AFM) as described previously [13]. All images were obtained at room temperature using a Multimode-Nanoscope V (Veeco, Santa Barbara, CA) operating in tapping mode with an etched silicon Probe model Arrow-NCR-50 Nano World (cantilever resonance frequency: 258 kHz, Force constant 42 N/m; tip radius 5–10 nm). Typical scan rates were 1–1.5 Hz.

### Other analytical methods

In order to ensure freshly, natively folded states, proteins were dissolved in ~2 M GndHCl and extensively dialyzed right before each experiment. Protein content was quantified by the Bradford technique [26], or by measuring optical density in a Bio-Rad spectrophotometer (Hercules, CA), using an extinction coefficient of 32,430 M^-1^cm^-1^ at 280 nm. Unless otherwise stated, results are representative of three independent experiments. Results are means ± standard error of at least three samples.

## Results

### Stability and conformation as a function of pH

**Fig 1:**
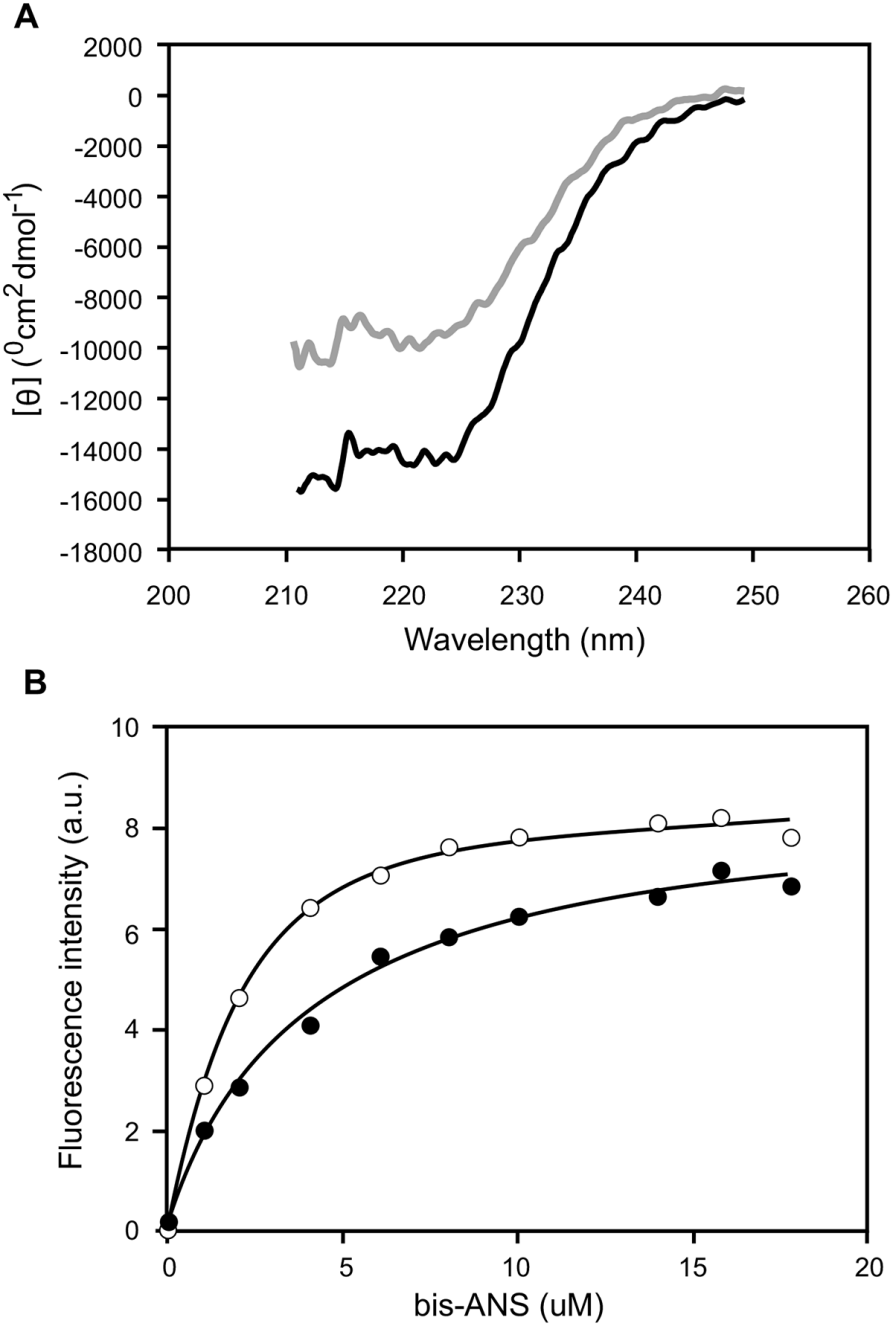
Spectroscopical characterization of protein structure. Wt and Arg173Pro variants (0.1 mg/mL) were dissolved in citrates phosphates McIlvaine’s buffer, pH 7.4. A) Far-UV circular dichroism. Dark and light grey lines correspond to wt and Arg173Pro proteins, respectively. B) Proteins were incubated with increasing concentrations of bis-ANS. The probe was excited at 360 nm, and the emission recorded at the wavelength of maximum fluorescence for this probe (488 nm). Filled and open circles represent the experimental data points for wt and Arg173Pro, respectively.

In order to characterize the effect of the substitution of an Arg residue by a Pro in the protein structure and stability, we set out to analyze whether this variant exhibited structural differences in the native state as compared with the wt protein. The far-UV circular dichroism (CD) spectra reveals a ~ 35% reduction in the secondary structure content of Arg173Pro with respect to the wt form (Fig 1A). A small but significant change in Trp environment was estimated by the analysis of the quenching effect with acrylamide. A significantly higher Stern-Volmer constant (K) measured for this mutant indicates an enhanced exposure of Trp residues to the solvent, as compared to the wt form (Table 1). In order to get further insight into the tertiary structure, proteins were titrated with bis-ANS, a dye which has been shown to be sensitive to hydrophobic surfaces and flexible structures [18]. The high fluorescence quantum yield associated to the binding of this probe supports previous notions of a spatial organization of the wt protein as a molten globule-like structure in the native state [15,18,19,23]. In addition, the Arg173Pro variant exhibits a higher quantum yield of the bound probe (Fig 1B), suggesting some difference in the spatial arrangement of the proteins. Together, these results suggest that the substitution of an Arg by a Pro induces conformational changes of the N terminal domain of apoA-I (where the Trp residues are located) that results in higher exposure of the Trp residues and of a hydrophobic protein surface to the aqueous medium.

**Table 1:** Fluorescence characterization of wt and Arg173Pro forms at pH 7.4 and 5.0.

Protein variants	K (M^-1^)^a^	ΔG° denat ^b^ (kcal/mol)	[GndHCl]_1/2_ ^c^	Scattering ^d^	E^e^	bis-ANS /Trp^f^
**pH 7.4**						
wt	5.58 ± 0.42	2.5 ± 0.3	0.9 M ± 0.2	1.05 ±0.32		
Arg173Pro	7.81 ± 0.91	N/A	0.5 M ± 0.3	2.17±0.38	0.23± 0.03	1.35± 0.41
**pH 5.0**						
wt	5.78 ± 0.87	2.4 ± 0.3	0.9 M ± 0.3	2.20±0.08		
Arg173Pro	6.14 ± 0.80	N/A	0.5 M ± 0.2	6.23±2.23	0.38± 0.04	1.40± 0.42

^a^ Stern-Volmer constant for the quenching of Trp residues by acrylamide.

^b^ Free energy change of unfolding and

^c^ concentration at which half of the protein is unfolded, respectively, calculated from equilibrium unfolding curves, as described previously [18] and shown in Fig 2. N/A, not assayed

^d^ Light scattering of proteins (0.2 mg/mL) measured after 48 h incubation at 37°C with excitation and emission wavelengths set at 350 nm.

^e^ Fluorescence resonance energy transfer efficiency (E), calculated from Eq (2). F_DA_ and F_D_ are the intensity of Trp emission in the presence and in the absence of the acceptor molecule bis-ANS (excitation set at 295 nm and emission at 335 nm).

^f^ Intensity ratio of the fluorescence associated to bis-ANS and Trp in the samples described above, measured by direct excitation of each dye, as described in ‘Methods’.

**Fig 2:**
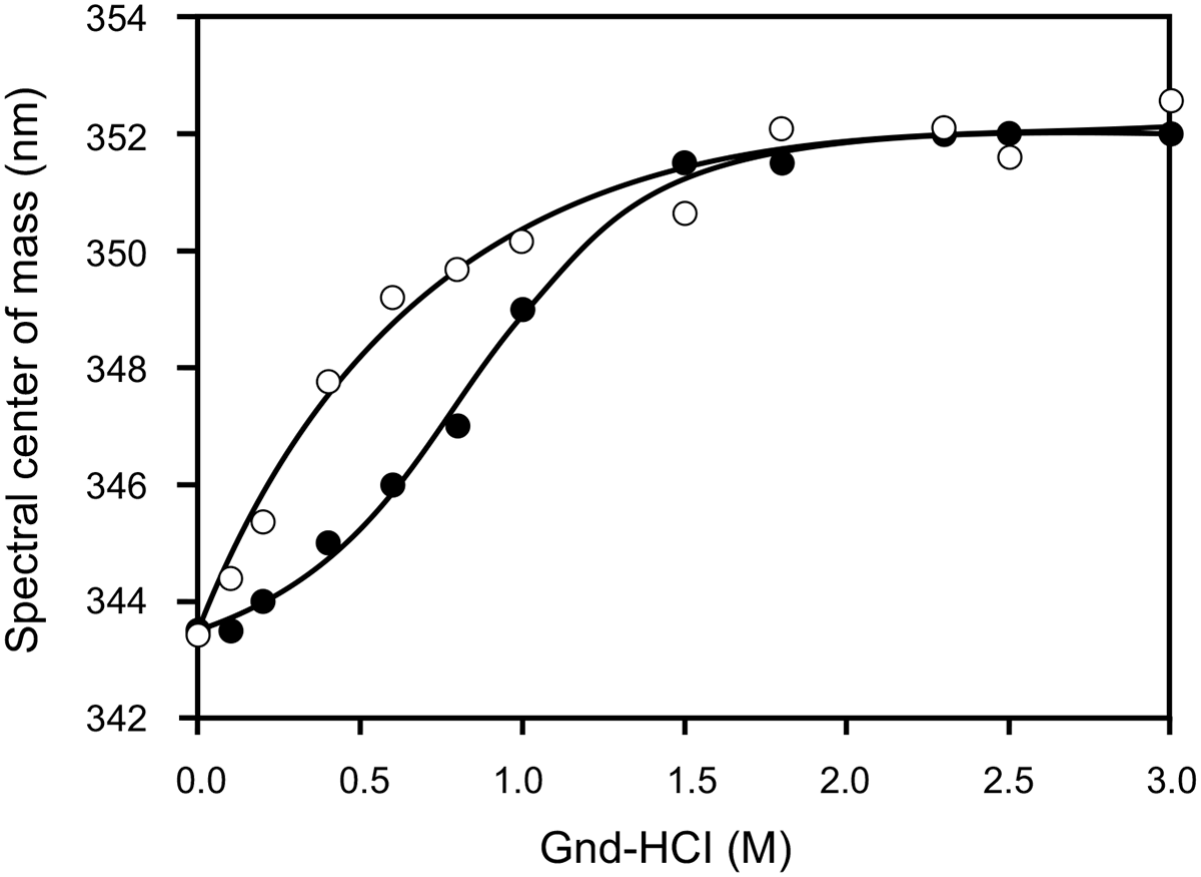
Chemical denaturation as followed by GndHCl titration. Proteins (0.1 mg/mL in citrates phosphates Mc Ilvaine’s buffer, pH 7.4) were incubated with increasing concentrations of GndHCl. Trp fluorescence emission spectra were obtained by excitation at 295 nm, and scanning the emission between 310 and 420 nm. With these data, the center of mass was calculated for each sample. Filled and open circles represent the experimental data for the wt and Arg173Pro variants, respectively.

Protein structure stability was investigated by equilibrium unfolding experiments, by following the intrinsic fluorescence emission at increasing concentrations of GndHCl. As it is well-known, chemical denaturation of wt apoA-I is described at pH 7.4 by a two-state denaturation model (Fig 2), from which the free energy of unfolding can be estimated [27]. In agreement with previously reported data, the measured ΔG° is 2.5 ± 0.3 kcal/mol, typical of a partially folded protein. Interestingly, the Arg173Pro denaturation behavior could not be fit to the two-state-model, thus preventing the estimation of ΔG°. Instead, even at the lowest GndHCl concentration assayed (0.1 M) a shift toward longer wavelengths is observed, indicating an enhanced exposure of Trp residues, giving hints on decreased stability. The apparent GndHCl concentration at which half of the protein is unfolded [GndHCl]_1/2_, is also significantly lower for this mutant (0.5 M) than for the wt variant (0.9 M (Table 1)).

### Binding lipid properties of the lipid-free variants

**Fig 3:**
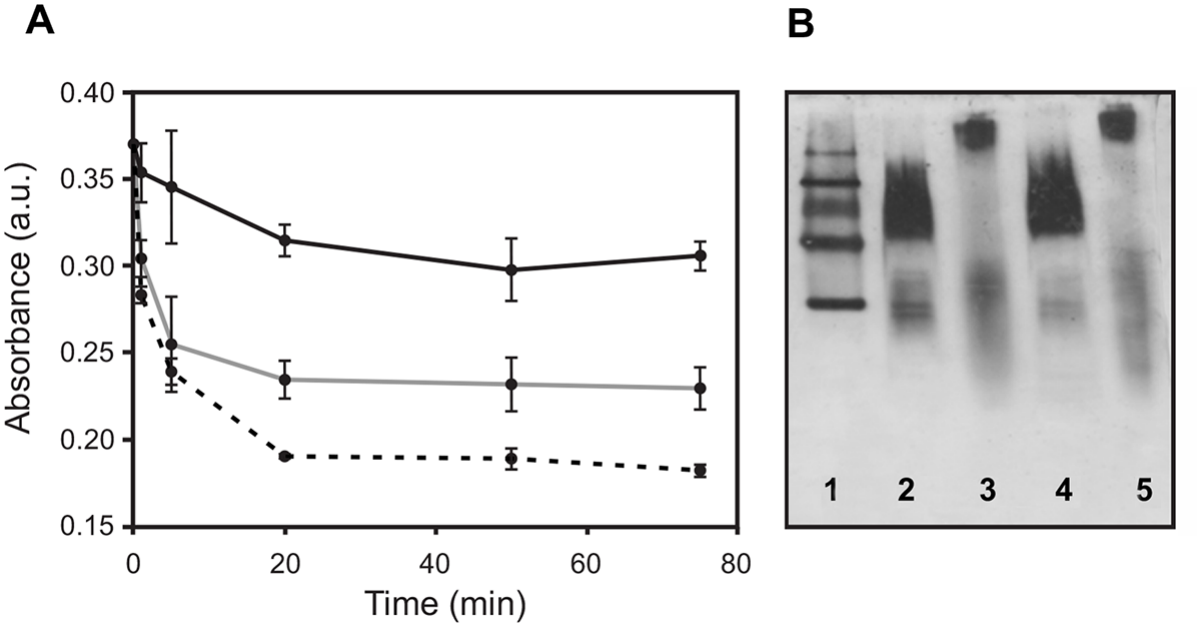
Characterization of spontaneous formation of lipid:protein complexes. A) Multilamellar DMPC liposomes were incubated at 24°C and Absorbance measured at 340 nm without (black) or with wt (dashed) or Arg173Pro (grey line) proteins at a 40:1 lipid to protein molar ratios. B) Native 4–24% PAGGE analysis of the final product after incubation of wt (lanes 2 and 4) or Arg173Pro (lanes 3 and 5) with DMPC at 24°C for 1 or 2 h, respectively. Lane 1 corresponds to High molecular weight standards 669, 440, 232, 140 and 67 kDa (Amersham Biosciences, UK).

In order to detect whether Arg173Pro keeps its functional role in lipid solubilization, we incubated this protein with DMPC vesicles at the lipid transition temperature (24°C) and compared its ability to form protein: lipid complexes by following the decrease in turbidity at 350 nm. As Fig 3A shows, lipid clearance was less efficient for Arg173Pro. In addition, the products after 1 or 2 h incubation were characterized by PAGGE (Fig 3B). As previously observed wt readily yielded a homogeneous population of around 230 kDa, indicating high efficiency to rearrange and to solubilize lipids. Instead the most of the Arg173Pro remained as lipid-free or lipid—poor protein and some amount recombined in higher size particles. This pattern was mantained after 24 h (results not shown).

### Formation of amyloid-like aggregates

**Fig 4:**
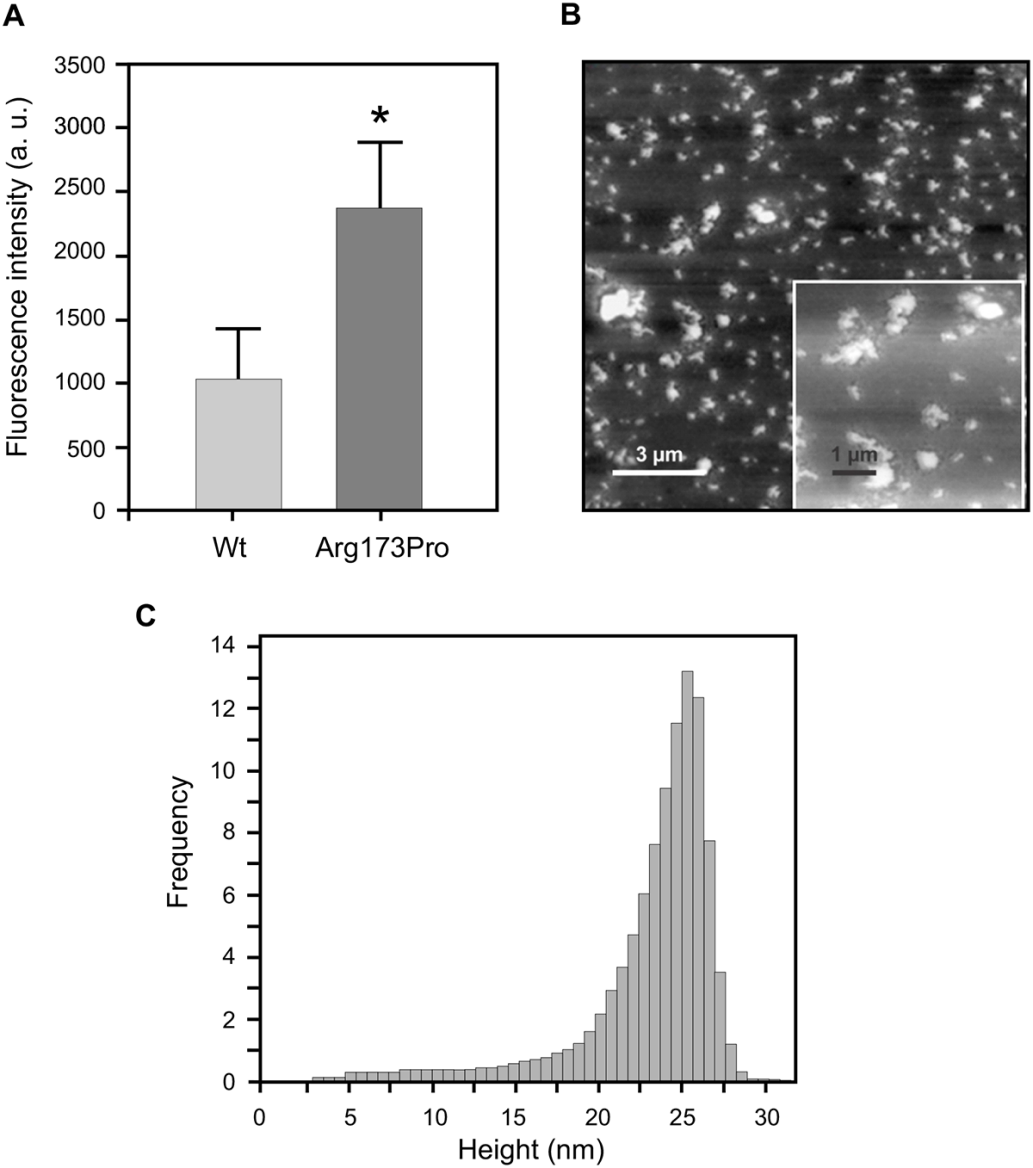
Characterization of Arg173Pro aggregates at pH 7.4. A) Binding of ThT to apoA-I. Proteins (0.2 mg/mL in citrates phosphates McIlvaine’s buffer, pH 7.4) were incubated for 48 h at 37°C and ThT added to each sample at a 1:1 molar ratio to protein. Fluorescence was quantified in the microplate reader at 480 nm (excitation set at 430 nm). Bars correspond to means ± SE. Statistically significant differences between experimental conditions were evaluated by Student t test. Symbol * denotes a difference with respect to wt at p<0.05. B) Characterization of the morphology of Arg173Pro aggregates after analysis of AFM images. Protein (0.5 mg/mL) was incubated for 24 h and loaded onto mica plates. Heterogeneous size oligomers covering the surface of the mica were predominant in the sample. The inset represents a frame at higher magnification, allowing us to observe elongated aggregates with a protofibrillar shape. Bars show the scale used in each case. C) Histogram distribution of the height of the oligomers obtained from measurements in the *z*-plane.

Then we analyzed the relative tendency of the Arg173Pro variant to aggregate even at physiological pH. The fluorescence quantum yield of ThT is very low in aqueous buffer and markedly increases upon binding to protein amyloids [28,29]. In agreement with previous data, even after 48 h incubation at pH 7.4, the wt form shows very low ThT associated fluorescence. Instead, fluorescence is significantly enhanced when ThT is bound to the Arg173Pro variant (Fig 4A). This evidence agrees with the formation of pro amyloidogenic protein complexes, which should be of larger size than wt, as suggested by higher light scattering values (Table 1). This tendency was demonstrated to be dependent on protein concentration and incubation time (results not shown). The characterization of protein aggregates by atomic force microscopy (AFM) indicates the main presence of aggregates of heterogeneous size (Fig 4B), and even the presence of some elongated structures detected at higher magnification (inset). In order to estimate the dimensions of the oligomers, their height was measured and represented in a histogram (Fig 4C). A wide, monomodal, slightly skewed distribution is observed, with most of the aggregates showing a height between 22 and 25 nm. This pattern is significantly different from that observed for the wt form assayed under the same conditions, in which a homogeneous, smaller size oligomer distribution is the characteristic feature [13]. Upon aggregation of the Arg173Pro variant, a decrease in the far UV-CD intensity is observed (especially at pH 5.0). Nevertheless, no conclusive information about the nature of the aggregates can be obtained due to the high proportion of native protein remaining soluble (result not shown).

### Effect of pH on protein structure, stability and aggregation propensity

**Fig 5:**
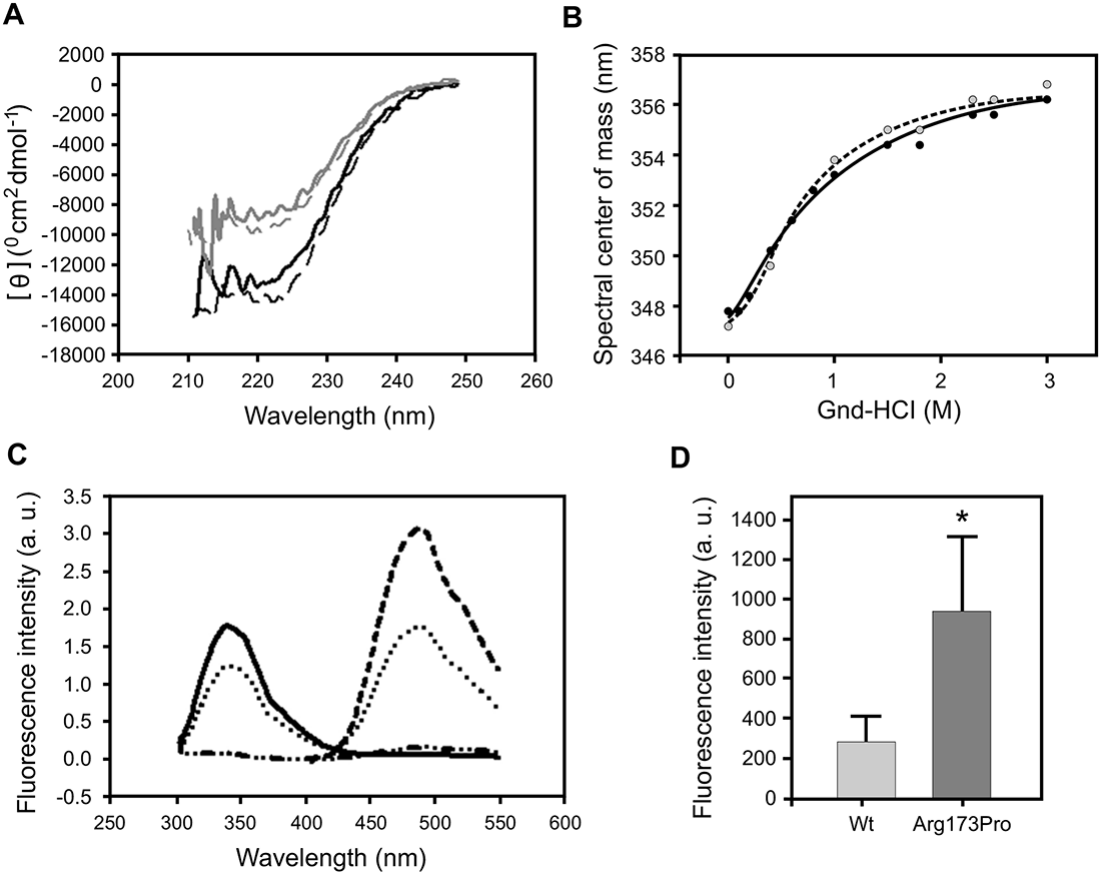
Protein structure, stability and aggregation tendency at pH 5.0. A) Proteins were dissolved in citrates phosphates McIlvaine’s buffer, pH 5.0 and far-UV circular dichroism measured as describe above. Dark and light grey solid lines correspond to the wt and Arg173Pro variants, respectively. To facilitate comparison, dashed lines correspond to both proteins measured at pH 7.4 (as shown in Fig 1A); B) Arg173Pro (0.1 mg/ml, pH 5.0) was incubated with increasing concentrations of GndHCl and the center of mass of the Trp fluorescence measured as described above (filled circles). Here again, to facilitate comparison, dashed lines correspond to the protein measured at pH 7.4 (as shown in Fig 2). C) Analysis of energy transfer from Trp to bis-ANS. The Arg173Pro variant was dissolved in citrates phosphates McIlvaine’s buffer (0.1 mg/mL) and Trp fluorescence measured (with excitation set at 295 nm, continuous line). Bis-ANS was added next and spectra measured (with excitation set at 295 nm, dotted line). Direct excitation of bis-ANS was achieved at 395 nm (dashed line). Dashed and dotted line represents bis-ANS excitation at 295 nm in protein-free buffer. Efficiency was calculated by measuring the intensity of Trp fluorescence at the wavelength of maximum emission (335 nm). D) Binding of ThT to apoA-I. Proteins (0.2 mg/mL in citrates phosphates McIlvaine’s buffer, pH 5.0) were incubated for 48 h at 37°C and ThT added to a 1:1 molar ratio to protein. Fluorescence intensity was quantified in the microplate reader at 480 nm (excitation set at 430 nm). Bars correspond to means ± SE. Statistically significant differences between experimental conditions were evaluated by Student t-test. The symbol * denotes a difference with respect to wt at p<0.05.

Our previous results indicate that a mild decrease in the local pH does not induce drastic changes on the wt protein structure under short time incubation at room temperature [15]. As a change in net charge is expected for the mutant protein (a positively charged Arg residue is replaced by a neutral Pro) we set out to analyze the influence of acidic pH on the conformation of this variant. The CD spectrum (Fig 5A) and the chemical denaturation profile (Fig 5B) of the Arg173Pro mutant are preserved and look almost indistinguishable from those observed at pH 7.4.

To get further information on the effect of pH on protein folding, we analyzed the efficiency of energy transfer from Trp to the acceptor dye bis-ANS. Efficient energy transfer is detected either by the decrease in the emission intensity of the Trp residues, or by the increase in bis-ANS fluorescence (Fig 5C). In order to quantify FRET efficiency Eq (2), we registered Trp fluorescence emission before (filled line) and after the addition of bis-ANS (dotted line). As shown in Table 1, the efficiency of energy transfer is 0.38 at pH 5.0, a value about 15% higher than that measured at pH 7.4 (0.23). Under this acidic condition, there is a small, albeit not significant, increase in the ratio of bis-ANS (by direct excitation) vs. Trp fluorescence emission (Table 1). The intensity measured was registered after stepwise addition of GndHCl. As it was observed before [13], bis-ANS fluorescence decreases sharply following a monotonous trend with increasing GndHCl concentration, a feature indicating the presence of a flexible structure with hydrophobic patches maximally exposed in the native state. The effect was similar at both pHs tested. We then proceeded to analyze the propensity of proteins to aggregate at pH 5.0. After 48 h incubation at 37°C, the Arg173Pro variant exhibits a higher level of ThT emission intensity than the wt protein (Fig 5D) and scatters more light (Table 1).

### Ligand binding

**Fig 6:**
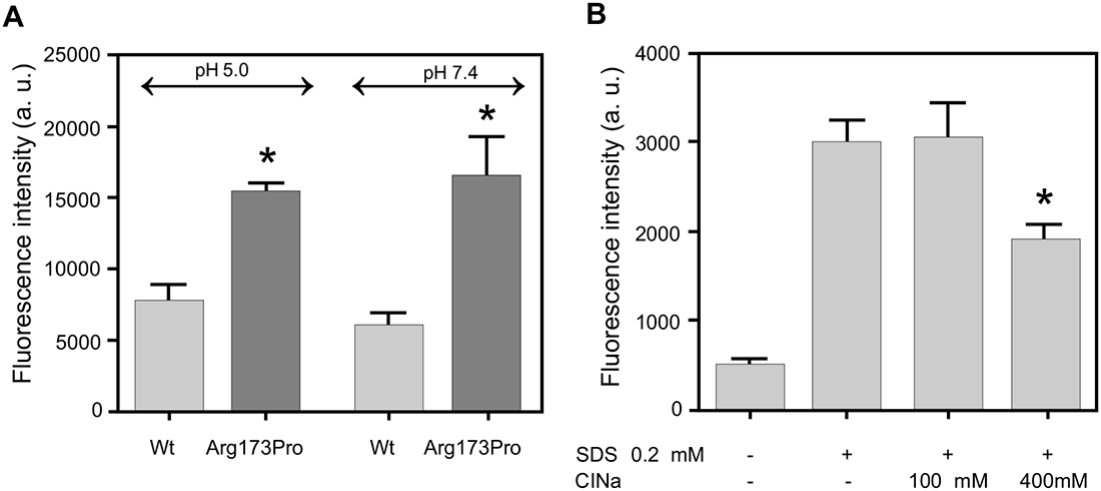
Protein binding to SDS. A) The wt and Arg173Pro variants (0.2 mg/mL) were incubated (either at pH 7.4 or 5.0) in the absence or in the presence of 0.2 mM SDS. After 48 h at 37°C ThT was added to a 1:1 molar ratio to protein. Fluorescence intensity was quantified in the microplate reader at 480 nm (excitation set at 430 nm). Statistically significant differences between experimental conditions were evaluated by ANOVA followed by Tukey’s test. Bars correspond to means ± SE. The symbol * denotes difference with respect to wt at p<0.01. B) The effect of salt concentration was tested by incubating Arg173Pro (0.2 mg/mL) at pH 7.4 in the absence or the presence of 0.2 mM SDS plus 0, 100 and 400 mM ClNa. Incubation was performed as in A). The symbol # denotes difference with respect to the protein in the absence of ClNa at p<0.01

The conversion of soluble, natively folded proteins into misfolded aggregates usually involves structural transitions leading to the partial or complete disruption of the native structure. Along this pathway, it has been suggested that weak interactions of the protein with molecules belonging to the micro-environment could favor the shift of the equilibrium toward a pathological conformation. SDS mimics some characteristics of biological membranes, and it has been described as an inducing agent for the formation of fibrils from different peptides and proteins when used below the critical micellar concentration (CMC: 0.7 mM) [30,31]. Within this frame, we analyzed the effect of SDS on the aggregation of wt protein and its pathological mutant, and the effect that the decrease in pH could bring about in promoting this interaction. Fig 6A shows that incubation of each protein with 0.2 mM SDS (either 5.0 or pH 7.4) elicits ThT binding, this effect being more pronounced for the Arg173Pro variant. This binding decreases in the presence of NaCl (Fig 6B) and is more notorious at pH 5.0 (not shown).

**Fig 7:**
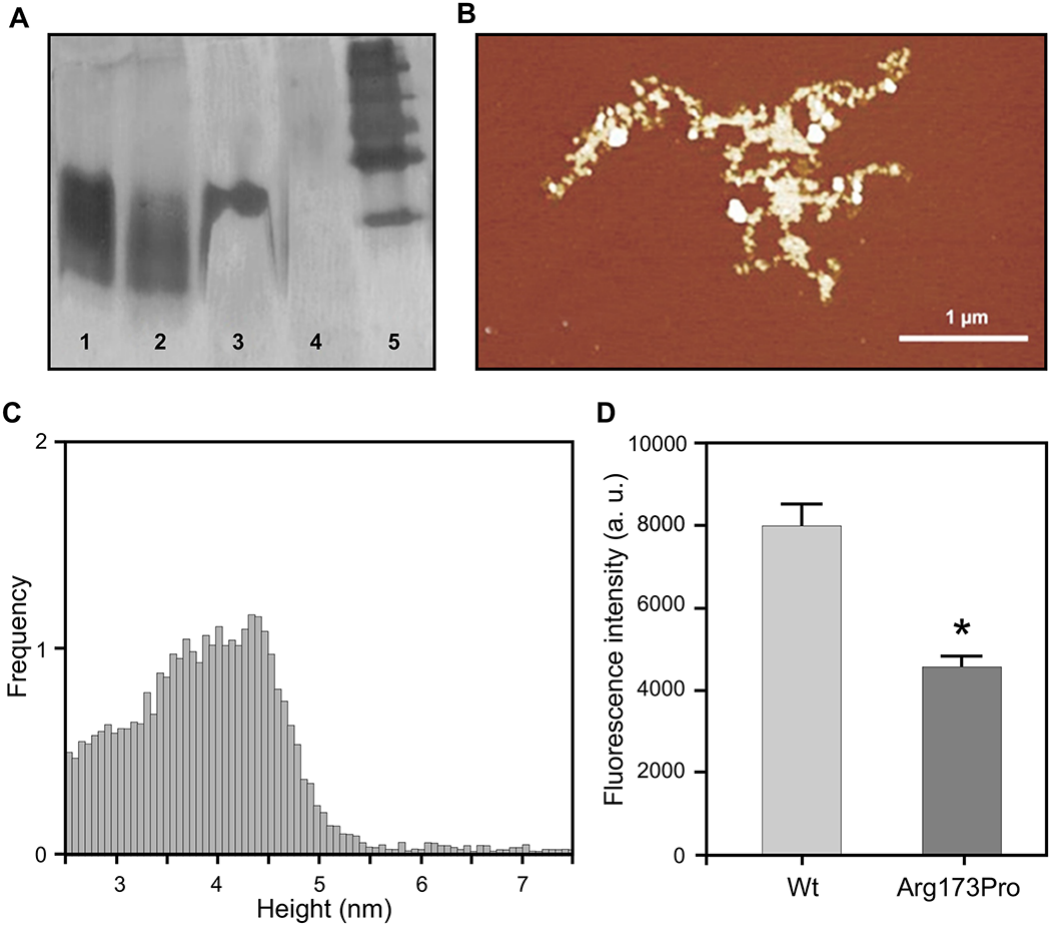
Characterization of heparin binding to apo A-I variants. Polyacrylamide gradient gel electrophoresis (PAGGE, 4–25%) under native conditions. wt (lane 1) and Arg173Pro variants (lane 2); lanes 3 and 4 correspond to wt and Arg173Pro variants plus heparin (added at a 2:1 heparin to protein molar ratio), after 48 h incubation at 37°C at pH 7.4. B) Characterization of the morphology of Arg173Pro aggre**g**ates with he**p**arin. Analysis of images observed under AFM. Proteins (0.5 mg/mL) were incubated for 24 h and loaded onto mica plates. The bar shows the scale indicated in the image. C) Distribution of the height of oligomers resulting from the measurement in the *z*-plane. D) Proteins (0.2 mg/mL in citrates phosphates McIlvaine’s buffer, pH 5.0) were incubated for 48 h at 37°C in the presence or in the absence of heparin at a 1:1 molar ratio. Binding of ThT was measured as described above. Bars correspond to means ± SE. Statistically significant differences between experimental conditions were evaluated by Student t-test. Bars correspond to means ± SE. The symbol * denotes the difference with the same protein without heparin at p<0.01.

Next we set out to check the effect of heparin as a putative ligand. We have previously shown that the wt form does not significantly interact with heparin at pH 7.4 [13]. In order to compare this behavior with that of the mutant, we incubated the Arg173Pro variant with heparin at this pH and analyzed the possibility of complex formation by native gradient gel electrophoresis (PAGGE) (Fig 7A). As expected, lanes containing proteins without heparin (lanes 1 and 2) showed bands migrating about a position consistent with the MW expected for the monomers. When the wt form was incubated in the presence of heparin, most of the intensity remains associated to this band (lane 3), confirming that no significant interaction occurs under this condition. By contrast, the incubation of the Arg173Pro variant with heparin for 48 h results in the disappearance of the monomer band (lane 4), thus indicating the formation of larger complexes. In order to get further insight into this phenomenon, we compared this product under AFM. In this case, we could detect aggregates that were about 5 nm high, more extended and more amorphous than those of the protein in the absence of heparin (Fig 7B and 7C).

Finally, we analyzed the influence of low pH on the efficiency of proteins-GAGs interaction. Due to the negative charge of GAGs, binding to proteins is expected to be more efficient at lower pH as the protonation of His residues results in enhancement of the net positive charge. We have previously confirmed that finding with the wt form [15]. Here, when incubating the apoA-I variant and the wt form at pH 5.0, even though both proteins show a high level of ThT binding, the yield in fluorescence was higher for wt than for the Arg173Pro mutant (Fig 7D).

## Discussion

The tale of interactions among apoA-I and ligands in circulation is largely studied in order to understand the main role of this protein in lipid homeostasis. However, little is known about the influence of the surroundings on protein misfolding and/or malfunction. We have previously suggested that a pathological landscape associated to inflammation could elicit the pro-amyloidogenic potential of the wt protein, as it is the case that could occur in the atherosclerotic plaque [9]. Nevertheless, the fact that severe amyloidoses are associated with point mutations in the protein sequence indicates that substitution or deletion of single amino acids could be critical to determine a pathway leading to defects in protein folding. Previous reports of our and other groups indicate that the structural features determining apoA-I amyloidogenicity are not strictly shared by all the mutants described in patients [13].

With this frame in mind, we characterized here structural consequences that could determine the pathogenicity of mutant Arg173Pro. As it was discussed above, substitution of an Arg by a Pro residue occurs in the so-called ‘hot spot’ domain of apoA-I, meaning that the disruption of this domain must bear a drastic effect on protein function or metabolism. The helix in which mutation occurs has been involved in lipid binding and lecithin cholesterol acyl transferase activity [32]. Our lipid-binding assay supports an impaired role of the protein to efficiently solubilize lipids from a bilayer, which is in agreement with the fact that HDL cholesterol levels of patients heterozygous for the mutation are low [14]. If this is the case, consequence could be not only a loss-of-protein function but in addition it could result in relatively higher amount of lipid-free or lipid-poor protein which is more sensitive to be modified by partners in the micro environment.

### Effect of destabilization of the native state on protein aggregation

For many amyloidogenic proteins, destabilization of the native globular state by mutations or by modification of the environment is highly correlated with the formation of amyloid fibrils, suggesting that the perturbation of the native folding is the critical event triggering amyloid formation [29,33,34]. Nevertheless, the surprising fact that the most destabilizing TTR mutants, Asp18Gly and Ala25Thr, are not the most pathogenic [35], indicates that the proposed correlation between stability and pathogenicity might indeed exist, but it should not be taken as a *sine qua non* condition. In support of this, it has been recently shown that stability is not a key event triggering amyloidogenicity of two natural mutants of apoA-I modified in the N-terminus [36]. Along this line of thought, we set out to determine whether a correlation could be found between the Arg173Pro propensity to misfold and its stability. The natural variant protein shows under physiological pH significantly lower (and less cooperative) chemical denaturation stability than the wt form, together with a partial loss of secondary structure, which might contribute at least in part to the higher aggregation tendency of the mutant. The red shift in Trp fluorescence of the Arg173Pro mutant, even at the lowest guanidinium concentration assayed, and a higher susceptibility to fluorescence quenching of these residues, indicate that the N-terminus of apoA-I (which holds in average the four natural Trp), is more solvent exposed in the mutant. Based on the recently obtained crystal structure of the C-terminal truncated apoA-I [37], Gursky et al proposed that either in the monomer or the dimer arrangement, lipid-free apoA-I structure is stabilized by two symmetry-related four-segment bundles at the opposite ends. They suggested that natural amyloidogenic mutations (comprising residues 44–120 and 154–184) occur in key positions in the helical bundle that disrupt this arrangement. The introduction of a Pro in the place of an Arg in position 173 should induce a kink in the helical sequence repeat 7 (residues Pro165−Gly185), which is expected to disorganize the interactions of this helical segment with the juxtaposed segment from repeats 2 and 3 [37]. If this is the case, it could be proposed that Trp 72 (in the helix 2), could be shifted from the native conformation in the protein structure. Interestingly, the N-terminus’ propensity to form amyloid structures was previously described [38] but in addition, the Trp 72 is comprised in one of the four hot spots predicted in apoA-I by applying AMYLPRED2 (peptides 14–22, 53–58, 69–72 and 227–232) [36]. Incubation of this variant under mild low pH does not seem to drastically contribute to protein stability and folding. Even though small structural changes were observed in the Trp environment after incubation of Arg173Pro at pH 5.0 (by quenching and FRET) the ThT associated fluorescence was about ~ 2.5–3 times higher than the intensity observed at pH 7.4, which is similar to the ratio measured for the wt under the same conditions.

### Lessons learnt from the binding of ligands

It has been previously shown that the lipid microenvironment could induce a conformational shift of apoA-I or peptides derived from terminal domains [39,40]. The presence of negatively charged lipids in the plasma membrane is a common hallmark associated to atherosclerosis or other local chronic inflammatory events inducing apoptosis. If this were the case, proteins could be exposed to local acidification in the interstitial fluid [41], which in turn could elicit the conformational shift of apoA-I into a pathologically misfolded state. In order to consider this hypothesis, we first checked the aggregation tendency of apoA-I variants in the presence of the negatively charged SDS, confirming an increased tendency to form amyloid-like aggregates either at pH 5.0 or 7.4. The headgroup of this amphiphile could participate in electrostatic binding with positive charges of the protein, which is in agreement with the loss of ThT binding observed in the presence of high concentrations of ClNa (Fig 6A). Nevertheless Arg173Pro variant was more efficient to give rise to pro-amyloidogenic complexes at both pH conditions assayed in spite of the loss of a positive charge (replacement of an Arg by a Pro). Thus, it is likely that, as it was suggested for the aggregation of other proteins as synuclein [42] and peptides [43], that alkyl chains of SDS could stimulate aggregation, perhaps by association with the non polar phases of the amphipatic helices, forcing intermolecular interactions.

**Fig 8:**
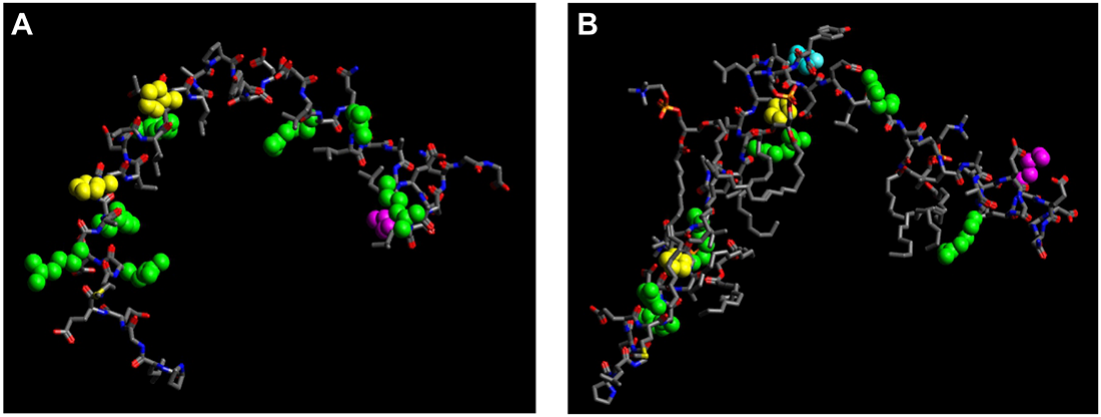
Model of the apoA-I peptide probably involved in the heparin binding site. (A) A model was built by using the Swiss-model database (www.expasy.org). The sequence loaded involves residues 143–186 in the wt form (PLGEEMRDRARAHVDALRTHLAPYSDELRQ**R**LAARLEALKENGG). Ball-and-stick representation was obtained with the Avogadro’s software. Positively charged residues are as follows: Arg are in green, His, in yellow and Lys, in pink. (B) Same for the Arg173Pro variant (PLGEEMRDRARAHVDALRTHLAPYSDELRQ**P**LAARLEALKENGG). The Pro residue incorporated with the mutation is shown in light blue.

Heparin and other glycosaminoglycans (GAGs) have been shown to interact with a wide variety of molecules, modulating numerous biological processes and to induce fibril or other misfolded conformations from many proteins [44]. In this context, we previously shown that wt apoA-I does not significantly bind heparin under physiological pH, but through a putative site generated under acidic pH following protonation of His 155 and 162 in the class A α-helix 6 [15]. When analyzing the Arg173Pro mutant, the ThT associated fluorescence was relatively decreased in the binding to heparin with respect to the wt protein at pH 5.0, a fact that could be explained by a lower efficiency to bind this GAG (Fig 7D). But especially interesting we believe it is the fact that, instead, the mutation induces a significant formation of complexes at pH 7.4 (Fig 7A and 7C). In order to find a possible explanation for this behavior, we built a model of the sequence of apoA-I involving helix 6 and 7 (Fig 8A). Arg 173 is located approximately in the middle of the putative helix 7 (comprising residues 165–186) and the helical wheel model predicts this residue to be facing the hydrophobic side of the amphipatic helix. Gursky et al proposed [37] that replacement by Pro (in light blue in Fig 8B) places a polar group in the bottom hydrophobic cluster and disrupts the native conformation around this region and -as a consequence- a significant alteration of the subtle interactions with neighboring helices that help to stabilize the entire molecule. By using the SOPMA secondary structure predictor software (www.expasy.org) for this region of apoA-I, the introduction of the Pro should induce a random coil segment of 4 amino acids disrupting the alpha helix. If this point mutation affects heparin binding, we could suggest that helix 7, in which the mutation occurs may be implicated in the binding motif involving helix 6 under acidic condition. The loss of the local positive charge, plus the disrupting effect of the Pro residue, could partially disorganize the spatial arrangement that is created under low pH, thus causing the decrease in ThT binding observed for the complex (Fig 7D).

On the other hand, the inclusion of the Pro residue results in the formation of a protein-GAG complex at pH 7.4 (Fig 7A and 7C). We can speculate that the disruption of helix 7 allows the exposure of cryptic residues that could potentially interact with the GAGs constituting a second binding motif. These helices contain several positive residues that could participate in this interaction: 3 Arg residues are aligned in helix 6 [15] and -in addition- helix 7 contains 3 positive residues as well (Arg 171, Arg 177 and Lys 182: Fig 8). All in all, we believe that the findings presented here offer new clues that help to understand pathological pathways, suggesting specific binding of the mutants to GAGs in a physiological context.

Along this line of evidence, examination of a heart specimen from a patient diagnosed with amyloid cardiomyopathy revealed substantial heparan sulfate accumulation in TTR amyloid deposits [45]. It is known that GAGs play complex functions in cell events as diverse as proliferation or aging [46,47]. In addition, it was recently proven that low molecular weight dermatan sulfate modulates endothelial cell proliferation and migration [48]. It is worth noting that scientific evidence supports that GAGs could participate in the amyloidogenesis *in vivo*, perhaps even in a protective role, by conversion of proteotoxic soluble oligomers into less toxic amyloid fibrils and related cross-β-sheet aggregates [49]. In our knowledge, it is not yet known the structure of apoA-I variants that could be more toxic inducing organ damage. Further research needs to be carried out in order to get a deeper insight into the cross-talk of apoA-I variants and ligands in circulation.
